# The effect of two remote exercise programs on cardiorespiratory fitness, cardiac function, and vascular health in patients with breast cancer

**DOI:** 10.14814/phy2.70787

**Published:** 2026-02-17

**Authors:** Nathan R. Weeldreyer, Charles C. Ellison, Mckenzie B. Mabalot, Cheyanne E. Helms, Zachariah B. Nealy, Zachary S. Leicht, Antonio Abbate, Christiana M. Brenin, Patrick M. Dillon, Trish Millard, Rebecca A. Krukowski, Jamie M. Zoellner, Siddhartha S. Angadi

**Affiliations:** ^1^ Integrated Cardiovascular Exercise Physiology (iCARE) Laboratory, College of health Sciences University of Alberta Edmonton Alberta Canada; ^2^ Department of Kinesiology University of Virginia Charlottesville Virginia USA; ^3^ Department of Public Health Sciences, School of Medicine University of Virginia Charlottesville Virginia USA; ^4^ Department of Medicine, Division of Cardiovascular Medicine University of Virginia Charlottesville Virginia USA; ^5^ Division of Hematology/Oncology, Department of Medicine University of Virginia Charlottesville Virginia USA

**Keywords:** cardio‐oncology, cardiorespiratory fitness, cardiovascular disease, exercise

## Abstract

Breast cancer is a common, survivable malignancy affecting women. With improved survival, the off‐target effects of chemotherapy, such as a decline in cardiorespiratory fitness and worse cardiovascular outcomes, have been recognized. Exercise training may help mitigate these effects. In this study, patients with breast cancer (*N* = 24) scheduled to undergo chemotherapy were randomized to either remotely administered high‐intensity interval training (HIIT) or moderate‐intensity exercise (MOD) based on ACSM guidelines for cancer survivors. HIIT involved three weekly sessions using the 4 × 4 protocol at 85%–90% peak heart rate (PHR), while MOD consisted of 150 min per week at 70%–75% PHR. Exercise training began 1–2 weeks before chemotherapy and continued throughout treatment. Baseline testing was conducted prior to chemotherapy and follow‐up testing 7–10 days after completion. Assessments included VO_2peak_, echocardiography, vascular function, and blood biomarkers. VO_2peak_ significantly declined in MOD (*n* = 8; 1.50 ± 0.23 to 1.27 ± 0.28 L/min; *p* = 0.013, *d* = 1.2), while remaining stable in HIIT (*n* = 7; 1.58 ± 0.24 to 1.52 ± 0.28 L/min; *p* = 0.445, *d* = 0.3), with a large between‐group effect size (*p* = 0.109, *d* = 0.9). NTproBNP levels significantly increased in MOD (104.3 ± 35.1 to 158.1 ± 56.0; *p* = 0.018), but not in HIIT. The study was underpowered due to greater than anticipated dropout to detect significant between‐group differences in VO_2peak_. These data have implications for the design of scalable exercise interventions in patients undergoing chemotherapy for breast cancer.

## 1 INTRODUCTION

Breast cancer is one of the most common types of cancer amongst women, with a lifetime risk of 1 in 8 women, and it is the second leading cause of cancer death (National Cancer Institute, 2025). With advancements in treatments, the survival rate for breast cancer continues to improve, with 5‐year survival rates exceeding 90% (American Cancer Society, 2025). Treatment for Stages I–IV breast cancer commonly involves systemic chemotherapy with a range of agents. As a result of increased survival, the off‐target effects of chemotherapy are becoming increasingly appreciated in breast cancer survivors (Kirkham et al., 2019). Off‐target effects include reduced quality of life (QoL), increased fatigue, reduced functional capacity (VO_2peak_), as well as cardiac, vascular, and skeletal muscle toxicity (Dickinson et al., 2017; Given et al., 2001; Lenneman & Sawyer, 2016; Mclaughlin et al., 2021; Sweeney et al., 2006). In fact, older breast cancer survivors are more likely to die from cardiovascular disease than from breast cancer (Patnaik et al., 2011).

These adverse effects are well known, and a significant amount of research has examined various ways exercise can be used to improve health in survivors (Speck et al., 2010). However, fewer studies have investigated how exercise during chemotherapy treatment can be used to preserve health. The recent BREXIT trial (Foulkes et al., 2023) demonstrated that cardiorespiratory fitness and cardiac reserve improved with a supervised exercise training program that transitioned to unsupervised exercise compared to usual care (recommendations to perform 150 min of moderate‐intensity exercise per week) during anthracycline‐based treatment in women with breast cancer. However, the study may be difficult to generalize and scale due to the supervision of exercise training during chemotherapy, which can be challenging for populations with low access to healthcare resources as well as a higher burden on health system resources. Rehabilitation referral rates and adherence are low for survivors of cancer (Hardcastle et al., 2018) in part due to poor access opportunities and lack of insurance coverage making them unaffordable. In‐person exercise rehabilitation is especially challenging for patients living in low‐resource rural environments.

The primary aim of this trial was to examine the feasibility and retention rate of conducting a fully remote exercise program for two exercise interventions: high intensity interval training (HIIT) vs. moderate intensity training (MOD) in women with breast cancer undergoing chemotherapy treatment (Helms et al., 2026). However, our secondary aim was to examine the effect of these remote interventions on important physiological outcomes such as cardiorespiratory fitness and cardiac function.

Exercise intensity plays an important role in the adaptations to VO_2peak_ and cardiac function seen following training in healthy volunteers without cancer (MacInnis & Gibala, 2017; Wisloff et al., 2009). However, data on the role of exercise intensity in patients with breast cancer undergoing active chemotherapy for preservation of VO_2peak_ is not well developed. Thus, the purpose of the current report was to compare these two remote interventions in attenuating reductions in cardiorespiratory fitness, cardiac function, and vascular function when initiated prior to and continued throughout chemotherapy treatment. We hypothesized that those randomized to the HIIT intervention would see a greater preservation of measures compared to those in the MOD intervention.

## 2 MATERIALS AND METHODS

### 2.1 Participants and study design

Patients enrolled for this study were recruited from the Breast Care Center at the University of Virginia Comprehensive Cancer Center. Eligible patients were those greater than 18 years old, had a breast cancer diagnosis (stages I–III or IV with minimal metastatic disease burden), spoke English, were given physician clearance for exercise training, and were prescribed one of the following chemotherapy regimens: Docetaxel/Cyclophosphamide (TC), Doxorubicin/Cyclophosphamide (AC), Docetaxel/Carboplatin/Trastuzumab/Pertuzumab (TCHP), Docetaxel/Carboplatin/Trastuzumab (TCH), or Pembrolizumab/Paclitaxel/Carboplatin followed by Pembrolizumab/Doxorubicin/Cyclophosphamide (KEYNOTE522 (Schmid et al., 2020)). Potential patients were excluded from the study if they had: (1) previous treatment with cardiotoxic chemotherapy; (2) clinically significant cardiac, renal, hepatic, hematologic, or pulmonary disease precluding exercise testing and training; (3) unstable angina or myocardial infarction within 4 weeks prior to treatment; (4) complex ventricular arrhythmias or New York Heart Association class IV symptoms; (5) symptomatic severe aortic stenosis; (6) recent pulmonary embolus; (7) acute myocarditis; (8) untreated high‐risk proliferative retinopathy; (9) recent retinal hemorrhage; (10) uncontrolled hypertension (systolic blood pressure >180 mm Hg or diastolic blood pressure >120 mm Hg); (11) severe baseline electrolyte abnormalities; (12) medication non‐compliance; (13) uncontrolled metabolic disease (diabetes with fasting blood sugar >300 mg/dL, thyrotoxicosis, myxedema); (14) or symptomatic peripheral vascular disease. This study was approved by the institutional review board at the University of Virginia and conducted in accordance with the Declaration of Helsinki. All patients provided written informed consent before partaking in any study procedures. This study was prospectively registered on clinicaltrials.gov (NCT05786014).

### 2.2 Exercise interventions

Patients were randomized into one of two exercise interventions: (1) high‐intensity interval training (HIIT) using a stationary, recumbent bicycle; (2) moderate‐intensity exercise (MOD) based on ACSM guidelines for cancer survivors which are similar to those used in the BREXIT trial (Foulkes et al., 2023) with remote monitoring. Exercise interventions were performed in an unsupervised, remote manner following two initial in‐person training sessions. Patients were given a smartwatch (Unite, Polar Electro, Finland) as well as a heart rate strap (H10, Polar Electro, Finland) in order to track their workouts and monitor heart rates throughout their exercise sessions. In addition, these data were uploaded and tracked via cloud‐based software (Polar Flow) for each patient. This allowed for real‐time data tracking and the delivery of personalized text messages throughout the intervention based on participants' current adherence. Patients randomized to the HIIT group were instructed to perform exercise sessions 3 days per week. Each session consisted of four 4‐min intervals at 85%–90% PHR separated by 3 min of active recovery at ~50% PHR. Sessions started with a 10‐min warmup and ended with a 5‐min cool down at ~50% PHR. In addition, patients were allowed to perform eight 2‐min intervals with 2‐min recovery, or five 3‐min intervals with 3‐min recovery as alternative options if they felt unable to complete the 4 × 4 protocol. Patients in the MOD group were asked to accumulate 150 min a week of moderate to vigorous physical activity via walking at 70%–75% of PHR in accordance with current American College of Sports Medicine Guidelines for patients with cancer (American College of Sports Medicine et al., 2021). Participants were allowed to exercise in bouts that added up to 150‐min/week in any way they liked.

To minimize barriers to exercise, participants in the HIIT group had recumbent bikes delivered and assembled in their homes, and those in the walking group were given gift cards to purchase running shoes. Interventions were guided by Social Cognitive Theory and evidence‐based behavioral change techniques (goal setting, action planning, problem solving, feedback, and self‐monitoring) (Helms et al., 2026). Patients in both groups attended two in‐person familiarization sessions to learn how to use the equipment, demonstrate proficiency in using them, and familiarize themselves with their exercise intensities. To support the participants' goals and adherence, the intervention also included one telephone‐based core session, personalized short message services (SMS), and telephone‐based stepped care sessions for problem solving. To monitor reasons for nonadherence, one SMS was sent out each week if exercise goals were not met, in which participants could choose from the following reasons: (1) chemotherapy side effects, (2) lack of time, (3) depression, (4) other, and (5) weather (MOD only).

### 2.3 Maximal exercise testing

All baseline testing occurred between 1 and 2 weeks prior to the initiation of chemotherapy. Post testing occurred 7–10 days following the last chemotherapy infusion. Cardiorespiratory fitness was assessed via maximal cardiopulmonary exercise tests. VO_2peak_ was determined by a ramp protocol conducted on a recumbent cycle ergometer (Ergoselect 600, Ergoline, Germany). Cycle ergometry was chosen over a treadmill in order to reduce potential discomfort associated with palmo‐plantar dysesthesia, a common side effect associated with chemotherapies (Kwakman et al., 2020). In addition, a recumbent ergometer was selected to reduce the strain on surgical sites that may come from the use of an upright ergometer. Following 2 min of resting data collection, a 5‐min warmup at 25 watts was performed. A ramp protocol was performed such that following the warmup the resistance continuously increased by 15 watts/min. Cadence was kept steady between 60 and 65 RPMs. Gas exchange data was collected as breath‐by‐breath data on the Vyntus CPX metabolic cart (Vyaire, Mettawa, IL). Continuous 12‐lead EKG was monitored throughout the test to measure heart rate and rhythm (CardioSoft, Vyaire, Mettawa, IL). VO_2peak_ was calculated as the average of the two highest VO_2_ values during the test. Peak heart rates obtained from this test were used to inform exercise prescriptions used in the intervention. In addition, ventilatory efficiency slopes (VE/VCO_2_ and VE/VO_2_) were calculated from the lines of best fit from the ramp portion of the exercise test. Oxygen uptake efficiency slope (OUES) was derived from plotting VO_2_ and log_10_VE (Baba et al., 1996).

### 2.4 Echocardiograms

Transthoracic echocardiograms (TTEs) were performed by trained sonographers following American Society of Echocardiography guidelines (ASE) (Mitchell et al., 2019) and read by cardiologists who were blinded to study allocation. Standard parasternal long and short axis views, apical 4 and 2 chambers views, apical long axis, and subcostal views were obtained. Main outcome measures included measures of left ventricular (LV) function and diastology. Specifically, systolic function was assessed using Simpson's biplane to determine LV ejection fraction (LVEF), 2‐dimensional speckle tracking was used to calculate LV global longitudinal strain (LVGLS) from apical 2, 4 and long axis views. In addition, cardiac output (CO) was estimated by multiplying heart rate by the product of the left ventricular outflow tract (LVOT) area and LVOT velocity (Zhang et al., 2019). LV diastology was assessed using mitral inflow velocities (E and A waves), mitral valve annular velocities (e′), tricuspid valve regurgitation velocity (TR VMAX), and left atrial volume index (LAVI). Values from these were used to calculate diastolic dysfunction (DD) grade based on ASE guidelines (Nagueh et al., 2016). Any missing values from the echocardiogram reports were calculated utilizing commercially available software (AGFA Enterprise Imaging) by a blinded member of the study team.

### 2.5 Flow mediated dilation

Endothelial function was measured via flow mediated dilation (FMD) of the brachial artery using 2‐dimensional and doppler ultrasound (EPIQ 7, Philips, Andover, MA or a usmart3300 ultrasound, Terason Ultrasound, Burlington, MA). FMDs were performed in accordance with current expert consensus recommendations (Thijssen et al., 2019) and as previously described (Tucker et al., 2021). Videos were analyzed within Vascular Tools 5 (Medical Imaging Applications, Coralville, Iowa).

### 2.6 Central hemodynamics

Both pulse wave analysis (PWA) and pulse wave velocity (PWV) were measured using the SphygmoCor Xcel system (AtCor Medical, Sydney, New South Wales, Australia). Data were collected as previously described (Tucker et al., 2021). Three trials were performed, and the two closest data points were averaged to obtain brachial and central blood pressure, augmentation pressure (AP), augmentation index (Aix), and augmentation index corrected for a heart rate of 75 bpm (Aix75).

### 2.7 Serum biomarkers

At baseline and post testing, blood samples were collected from the antecubital fossa or from an in‐dwelling port following an overnight fast. Samples were drawn into Ethylenediaminetetraacetic (EDTA) and serum separator tubes, centrifuged, and immediately stored in a −80°C freezer until analysis. In addition, standard of care blood draws were collected at the initial and final chemotherapy infusion visits via indwelling catheter, and an accredited laboratory was used to analyze changes in patient blood counts.

Stored samples were used to analyze markers of inflammation and cardiac damage via multiplex ELISA (MAGPIX, Luminex, Austin, TX). Specifically, we measured interleukin (IL)‐1β, IL‐6, and tumor necrosis factor (TNF) (HADK2MAG‐61K‐04 Millipore Sigma); N‐terminal prohormone of brain natriuretic peptide (NTproBNP) (HCVD1MAG‐67K‐01 Millipore Sigma); and C‐Reactive Protein (CRP) (HCVD3MAG‐67K‐01 Millipore Sigma) in accordance with manufacturer's instructions. Serum samples were run in duplicate, and trials with a greater than 20% coefficient of variance were excluded and rerun if necessary. In addition, samples with less than 70% recovery or greater than 130% recovery were also excluded per manufacturer recommendations. The average of all runs meeting the above criteria was used in the final analysis.

### 2.8 Statistical analysis

Statistical analyses were performed using SPSS Statistics v29.0.2.0 (IBM Armonk, NY). Independent samples *t*‐tests were used to assess for differences between groups at baseline. Changes (Delta) following intervention were calculated as post minus pretesting values. Normality of the deltas from pre‐ and post‐testing was assessed via Shapiro‐Wilks tests. To examine for between‐group differences, independent samples *t*‐tests were used to compare deltas between both groups for all outcome measures. In addition, paired samples *t*‐tests were run to assess for changes within‐group from pre‐ to post‐intervention. For measures that were not normally distributed, Wilcoxon‐signed rank tests and Mann–Whitney *U*‐tests were used. As this was a pilot study aimed to generate effect sizes to inform a large‐scale future trial, Cohen's *d* effect sizes were also calculated to examine the magnitude of effect from each study intervention. Data are presented as mean ± SD unless otherwise noted. Alpha was a priori set to 0.05.

## 3 RESULTS

### 3.1 Subject characteristics

**FIGURE 1:**
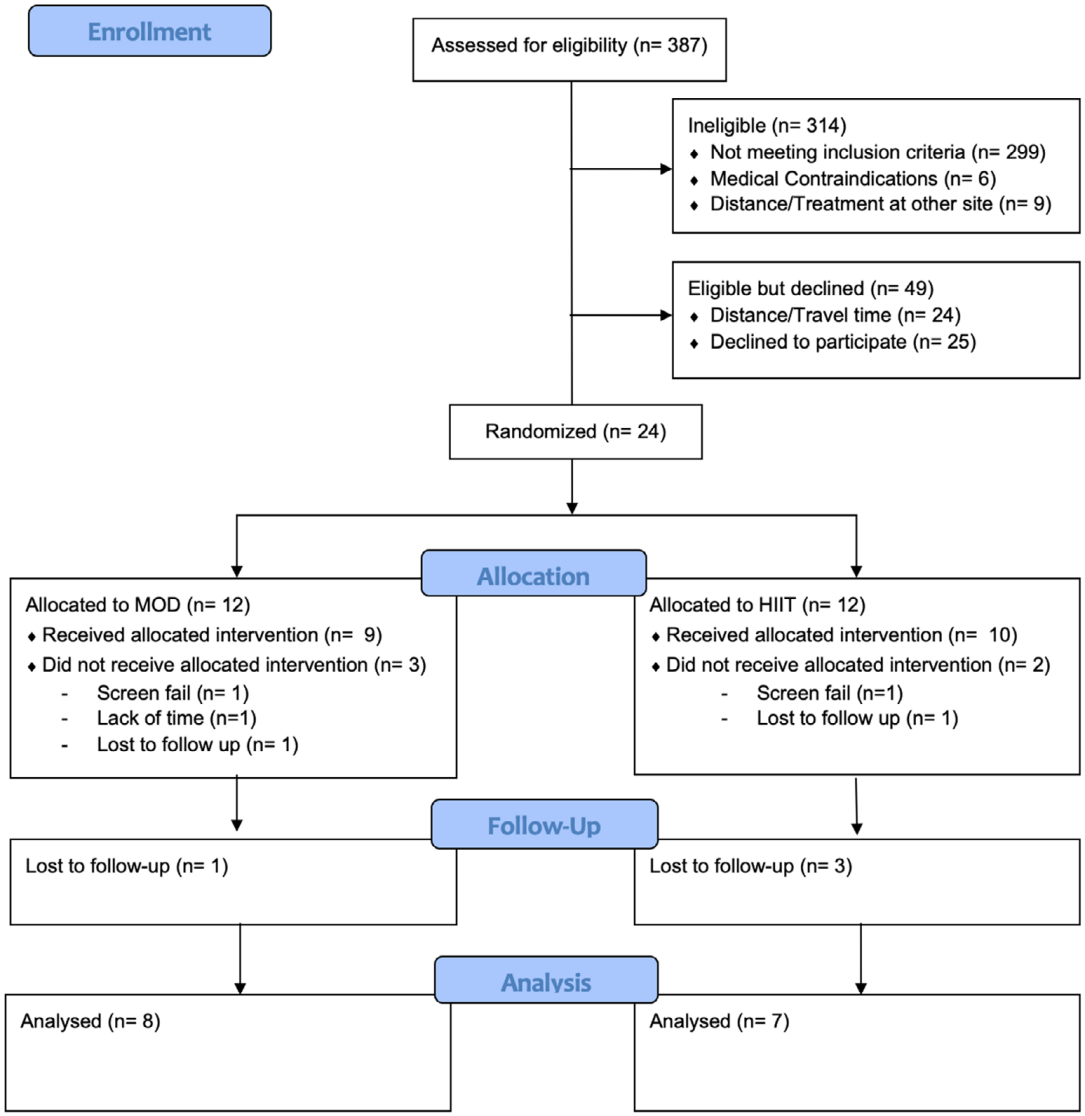
Prisma flow diagram of study recruitment and retention.

As shown in Figure 1, 24 patients consented and enrolled in the trial, and 15 patients completed all aspects of the study protocol (HIIT: *n* = 7, MOD: *n* = 8). Baseline characteristics and breast cancer treatments are presented in Table 1. At baseline, there was a significant between‐group difference in ejection fraction (HIIT: 67.5 ± 5.6% vs. MOD: 62.5 ± 2.5%, *p* = 0.047). No other differences were observed at baseline. While similar, due to different chemotherapy regimens the intervention duration for HIIT was 10.6 ± 3.8 weeks and 13.0 ± 6.2 weeks for MOD (*p* = 0.374). This difference was largely driven by one patient on the KEYNOTE522 therapy (which lasts 24 weeks).

**TABLE 1:** Patient demographics and characteristics.

	MOD (*n* = 8)	HIIT (*n* = 7)	*p*
Age	52.6 ± 11.2	55.3 ± 11.9	0.663
Weight (kg)	67.2 ± 9.1	78.7 ± 15.0	0.093
BMI	26.0 ± 4.9	28.9 ± 5.5	0.307
VO_2peak_ (L/min)	1.50 ± 0.23	1.58 ± 0.24	0.539
Ejection fraction (%)	62.5 ± 2.5	67.5 ± 5.6	0.047*
Race			
White	8	4	
Black/African American	0	3	
Breast cancer staging			
Stage I	3	3	
Stage II	5	3	
Stage III	0	1	
Receptor status			
ER and/or PR positive	3	4	
HER2 positive, ER, and PR negative	1	0	
HER2 and ER and/or PR positive	0	2	
Triple negative	3	1	
Chemotherapy regimen			
Doxorubicin + Cyclophosphamide	4	4	
Docetaxel + Cyclophosphamide	2	1	
Paclitaxel + Trastuzumab	1	2	
Keynote522	1	0	

Abbreviations: BMI, body mass index; ER, estrogen receptor; HER2, human epidermal growth factor receptor 2; PR, progesterone receptor.

* *p* < 0.05.

### 3.2 Exercise adherence

Patient adherence to the exercise programs was similar between the two arms: 61 ± 32% for HIIT and 64 ± 37% for MOD, respectively (*p* = 0.868). Principle cause of exercise noncompliance was chemotherapy related side‐effects. Full adherence data can be found elsewhere (Helms et al., 2026). No significant correlations were observed for exercise adherence and the delta of any primary outcome measure when assessed across groups (all *p* > 0.05).

### 3.3 Cardiorespiratory fitness

**FIGURE 2:**
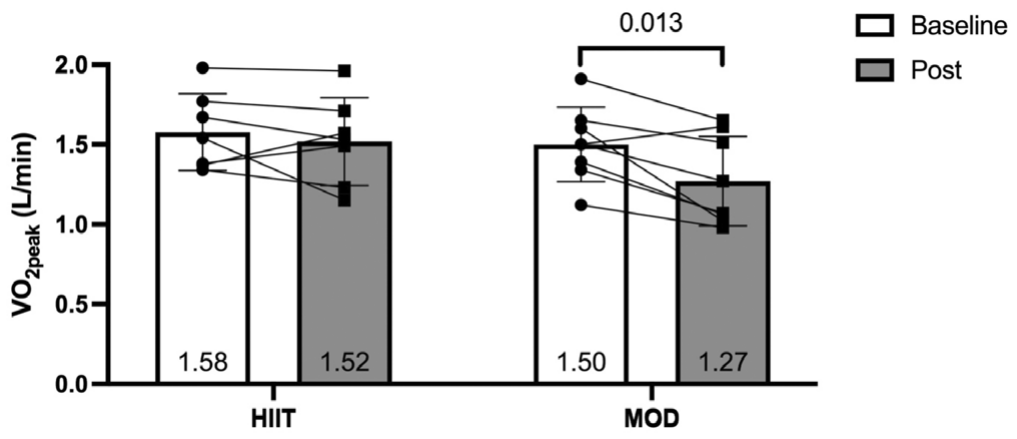
Changes in absolute VO_2peak_ following exercise training while undergoing chemotherapy treatment.

Those in the MOD group experienced a significant reduction in VO_2peak_ of 0.23 L/min from pre‐ to postintervention (1.50 ± 0.23 to 1.27 ± 0.28 L/min; *p* = 0.013, *d* = 1.2). In contrast, those in the HIIT group saw a nonsignificant reduction of 0.06 L/min (1.58 ± 0.24 to 1.52 ± 0.28 L/min; *p* = 0.445, *d* = 0.3) (Figure 2). While not statistically significant, the between‐group differences showed a large effect size (*p* = 0.109, *d* = 0.9). This resulted in a trend for reduction in peak power output in the MOD group (Table 2). Additionally, there were no significant within‐ or between‐group differences in VE/VCO_2_ slope (MOD: 31.6 ± 5.1 to 34.6 ± 8.3; *p* = 0.19, *d* = 0.5, HIIT: 33.2 ± 2.7 to 35.6 ± 4.8; *p* = 0.12, *d* = 0.7; between‐group: *p* = 0.86, *d* = 0.1). Of note, at the end of the trial, both groups had mean VE/VCO_2_ slopes >34, which is often used as a threshold beyond which patients with heart failure have worse outcomes (Myers et al., 2008). There was a significant reduction in OUES for MOD from pre‐ to post‐testing that did not occur in the HIIT group. While not significant, a large effect size for between‐group differences was observed (MOD: 1.63 ± 0.27 to 1.29 ± 0.26 L/min; *p* = 0.002, *d* = 2.5, HIIT: 1.57 ± 0.20 to 1.42 ± 0.23 L/min; *p* = 0.15, *d* = 0.6; between‐group: *p* = 0.14, *d* = 0.9).

**TABLE 2:** Changes in the primary outcome of VO_2peak_ and other CPET outcomes.

	MOD			HIIT			Between‐group
	Pre	Post	*p*	Pre	Post	*p*	*p*
VO_2peak_ (L/min)	1.50 ± 0.23	1.27 ± 0.28	0.013*	1.58 ± 0.24	1.52 ± 0.28	0.445	0.109
VO_2peak_ (mL/kg/min)	22.6 ± 2.5	19.0 ± 4.3	0.021*	20.7 ± 5.2	19.4 ± 4.3	0.167	0.151
Peak power output (watts)	110 ± 21	97 ± 28	0.089	125 ± 15	122 ± 27	0.625	0.319
%VO_2_ at VT	70.5 ± 8.5	82.6 ± 13.4	0.133	69.8 ± 4.9	73.2 ± 8.0	0.277	0.298
VE/VO_2_ slope	45.5 ± 7.9	55.4 ± 17.0	0.119	48.7 ± 9.1	51.0 ± 13.2	0.553	0.254
VE/VCO_2_ slope	31.6 ± 5.1	34.6 ± 8.3	0.19	33.2 ± 2.7	35.6 ± 4.8	0.12	0.86
OUES	1.63 ± 0.27	1.29 ± 0.26	0.002*	1.57 ± 0.20	1.42 ± 0.23	0.15	0.14

Abbreviations: OUES, oxygen uptake efficiency slope; slope VT, ventilatory threshold; VE/VCO_2_ slope, ventilation to VCO_2_ slope; VE/VO_2_ slope, ventilation to VO_2_.

* *p* < 0.05.

### 3.4 Cardiac function

**FIGURE 3:**
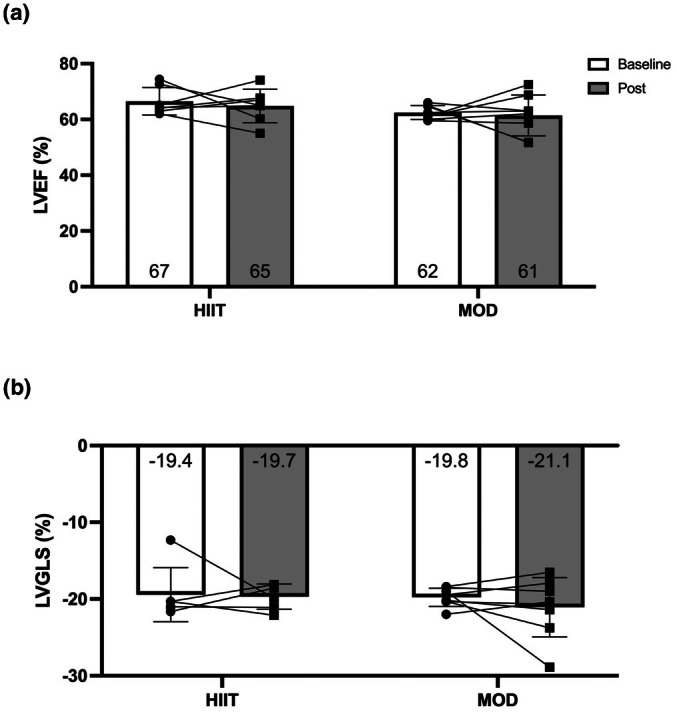
Measures of resting cardiac function in response to exercise training.

In assessing systolic function, no significant reductions were seen in ejection fraction (MOD: 62.5 ± 2.5% to 61.4 ± 7.3%; *p* = 0.749, *d* = 0.1, HIIT: 67.5 ± 5.6% to 62.6 ± 5.0%; *p* = 0.198, *d* = 0.7; between‐group: *p* = 0.427, *d* = 0.5) or in LVGLS (MOD: −19.8 ± 1.2% to −21.1 ± 3.9%; *p* = 0.380, *d* = 0.3, HIIT: −19.11 ± 3.8% to −20.0 ± 1.7%; *p* = 0.676, d = 0.2; between‐group: *p* = 0.851, *d* = 0.1) (Figure 3). A significant reduction was observed for e′ in the HIIT group, and there was no significant change observed for e’ in MOD or between groups (MOD: 11.08 ± 2.51 to 10.66 ± 3.21 cm/s; *p* = 0.549, *d* = 0.2, HIIT: 9.99 ± 2.87 to 8.33 ± 1.92 cm/s; *p* = 0.43, *d* = 1.1; between‐group: *p* = 0.206, *d* = 0.7). No other measures of diastolic function showed significant changes (Table 3). A trend for significant between‐group differences was observed for resting CO (*p* = 0.097) and a significant increase in the HIIT group (MOD: 4.8 ± 1.0 to 4.7 ± 1.7; *p* = 0.889, HIIT: 4.3 ± 1.1 to 5.3 ± 1.0 L/min; *p* = 0.004) owing to nonsignificant increases in heart rate and stroke volume (Table 3).

**TABLE 3:** Changes in the echocardiographic parameters.

	MOD			HIIT			Between‐group
	Pre	Post	*p*	Pre	Post	*p*	*p*
Ejection Fraction (%)	62.5 ± 2.5	61.4 ± 7.3	0.749	67.5 ± 5.6	62.6 ± 5.0	0.198	0.427
LV GLS (%)^a^	−19.8 ± 1.2	21.1 ± 3.9	0.674	−19.11 ± 3.8	−20.0 ± 1.7	0.893	0.622
E/A	1.18 ± 0.28	1.22 ± 0.50	0.699	1.4 ± 0.4	0.99 ± 0.3	0.081	0.059
Deceleration time (ms)	184 ± 41	176 ± 47	0.741	156 ± 60	175 ± 28	0.450	0.422
e′ (cm/s)	11.08 ± 2.51	10.66 ± 3.21	0.549	9.99 ± 2.87	8.33 ± 1.92	0.043*	0.206
E/e′	7.40 ± 1.70	7.95 ± 1.96	0.503	8.04 ± 2.21	9.17 ± 4.30	0.317	0.664
LAVI (mL/m^2^)	18.8 ± 8.2	23.1 ± 8.6	0.549	18.9 ± 9.4	28.6 ± 8.6	0.247	0.594
SV (mL)	64 ± 13	59 ± 19	0.487	65 ± 13	71 ± 15	0.270	0.224
Heart rate (BPM)	75 ± 6	80 ± 13	0.343	66 ± 9	77 ± 14	0.087	0.440
CO (L/min)	4.8 ± 1.0	4.7 ± 1.7	0.889	4.3 ± 1.1	5.3 ± 1.0	0.004*	0.097

Abbreviations: CO, cardiac output; E/A, ratio of early to late diastolic filling of the left ventricle; E/e′, ratio of early diastolic filling to mitral annular velocity; e′, mitral annular velocity; GLS, global longitudinal strain; LAVI, left atrial volume index; SV, stroke volume.

^a^ Indicates nonparametric test used.

* *p* < 0.05.

### 3.5 Vascular outcomes

No significant between‐group differences were observed for peripheral or estimated central blood pressures. There were trends for between‐group differences in peripheral diastolic blood pressure (DBP), central systolic blood pressure (SBP), and central DBP. Table 4 contains a full breakdown of vascular outcomes. A significant difference was observed in Aix75 between groups as well as within the MOD group (MOD: 26.4 ± 9.9% to 22.6 ± 9.4%; *p* = 0.027, *d* = 1.0, HIIT: 26.2 ± 7.9% to 28.2 ± 10.7%; *p* = 0.402, *d* = 0.3; between‐group: *p* = 0.04, *d* = 1.2). No significant differences were found within the HIIT group for any vascular outcome. In contrast, those in the MOD group had significant reductions in central SBP (111 ± 5 to 104 ± 4 mmHg; *p* = 0.008, *d* = 1.3). There were also trends for reductions in peripheral SBP (120 ± 6 to 116 ± 6 mmHg; *p* = 0.067, *d* = 0.8), DBP (78 ± 2 to 74 ± 6 mmHg; *p* = 0.093, *d* = 0.6), and Aix (29.0 ± 13.2 to 20.8 ± 13.2%; *p* = 0.062, *d* = 0.8) for those in MOD.

**TABLE 4:** Changes in blood pressures and measures of arterial stiffness.

	MOD			HIIT			Between‐group
	Pre	Post	*P*	Pre	Post	*p*	*p*
SBP (mmHg)	120 ± 6	116 ± 6	0.067	131 ± 14	132 ± 11	0.806	0.206
DBP (mmHg)	78 ± 2	74 ± 6	0.135	79 ± 8	81 ± 9	0.405	0.099
cSBP (mmHg)	111 ± 4.9	104 ± 4	0.008*	121 ± 14	122 ± 12	0.859	0.099
cDBP (mmHg)	78 ± 2	74 ± 6	0.093	80 ± 7	82 ± 9	0.356	0.073
Aix (%)	29.0 ± 13.2	20.8 ± 13.2	0.062	31.6 ± 6.9	31.5 ± 11.0	0.979	0.106
Aix75 (%)	26.4 ± 9.9	22.6 ± 9.4	0.027*	26.2 ± 8.0	28.2 ± 10.7	0.402	0.040*
PWV (m/s)	6.5 ± 0.8	6.1 ± 1.2	0.187	6.98 ± 0.61	6.64 ± 1.3	0.403	0.968
FMD (%)	12.2 ± 5.7	9.4 ± 2.9	0.378	9.9 ± 4.9	7.0 ± 3.9	0.063	0.979

Abbreviations: Aix, augmentation index; Aix75, augmentation index normalized to a heart rate of 75 bpm; cDBP, central diastolic blood pressure; cSBP, central systolic blood pressure; FMD, flow mediated dilation; PWV, pulse wave velocity.

* *p* < 0.05.

### 3.6 Biomarkers

**FIGURE 4:**
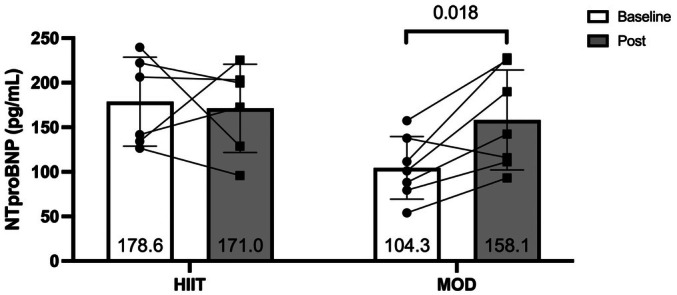
Changes in NTproBNP following exercise training.

Patients in both groups became anemic throughout the study. Hemoglobin was reduced significantly in both groups (MOD: 13.0 ± 0.6 to 10.7 g/dL ± 0.9; *p* = < 0.001, *d* = 2.2, HIIT: 12.4 ± 1.5 to 10.9 ± 0.9 g/dL; *p* = 0.015, *d* = 1.3; between‐group: *p* = 0.171, *d* = 0.8), and there was no significant difference between groups. No significant between‐ or within‐group differences were found in any marker of inflammation (all *p* > 0.05) (Table S1). A significant increase in NTproBNP was observed in the MOD group (104.3 ± 35.1 to 158.1 ± 56.0; *p* = 0.018), and no change was observed in the HIIT group (178.6 ± 49.9 to 171.1 ± 49.5; *p* = 0.793). This resulted in a trend for significant differences observed between groups (*p* = 0.075) (Figure 4).

## 4 DISCUSSION

The primary finding from this pilot trial was that there were no significant between‐group differences when comparing HIIT and MOD for preserving cardiorespiratory fitness (VO_2peak_) in women with breast cancer undergoing treatment with chemotherapy. However, a large effect size was observed when comparing between‐group differences (*d* = 0.9). In addition, there were no significant within‐ or between‐group differences when examining LV ejection fraction or LVGLS. However, there was a significant increase in resting CO in the HIIT group secondary to an increase in stroke volume and heart rate, which was not observed in MOD. While overall exercise adherence was moderate to low, primarily due to reported symptom burden during chemotherapy weeks, there were no differences in exercise adherence between groups.

The lower‐than‐expected adherence rate observed in the current trial may be an inherent limitation to remote exercise interventions prescribed for patients undergoing chemotherapy. For example, two supervised exercise programs for women being treated for breast cancer reported ~83% adherence on average (Jones et al., 2013; Lee et al., 2019), a 20% difference from the current trial. Throughout the intervention, patients received weekly personalized text messages, which included motivational support and, when low adherence was observed, asked about the primary reason for it. The largest reason for low exercise adherence was symptom burden during chemotherapy. This issue was addressed in the recent BREXIT trial (Foulkes et al., 2023), in which low‐intensity physical activity was prescribed the week of chemotherapy infusions, with intensity increased in the subsequent weeks. Since the purpose of the current trial was to explicitly compare the effects of moderate‐ versus high‐intensity exercise, this is an inherent limitation to the study design.

### 4.1 Cardiorespiratory fitness

The current study found no significant between‐group differences in VO_2peak_. However, while the MOD group saw a significant reduction in VO_2peak_, no such change was observed in the HIIT group. The current study is largely in agreement with previous trials in breast cancer patients receiving chemotherapy which show a preservation or improvement in VO_2peak_ with any prescribed exercise (Courneya et al., 2007; Foulkes et al., 2023; Hornsby et al., 2014; Mijwel et al., 2018). The majority of prior trials include a mix of exercise modes or intensities and cannot conclude superiority of any modality. A primary pathway by which chemotherapies elicit cytotoxicity is through increased reactive oxygen species (Yang et al., 2018). Exercise may counteract this in a tissue‐specific manner by increasing antioxidant capacity within metabolically active tissues (i.e., cardiac and skeletal muscle) (Assi et al., 2020), thereby contributing to the preservation of VO_2peak_. Indeed, previous animal models have shown exercise to be protective during doxorubicin treatment in cardiomyocytes and skeletal muscle (Dickinson et al., 2017; Kavazis et al., 2010). With this framework in mind, HIIT may lead to greater protection than MOD due to its greater ability to increase antioxidant capacity (Paramita et al., 2022). Our study is the first to directly compare the two exercise intensities and support this hypothesis. Larger, adequately powered studies should be performed to confirm this hypothesis and compare these changes to a true control group.

### 4.2 Cardiac function

There were no significant changes seen within or between HIIT or MOD for LVEF or LVGLS. This is a promising finding as it is well established that chemotherapy can lead to significant reductions in cardiac systolic function. However, previous clinical trials during anthracycline and trastuzumab therapies have been unsuccessful in showing differences in cardiac function with exercise compared to usual care (Hornsby et al., 2014; Jacquinot et al., 2022; Kirkham et al., 2017, 2018). This may be due to the fact that previous studies did not observe a decline in function within the usual care groups. Foulkes et al. reported a limited effect of exercise on resting measures of cardiac function when compared to usual care. However, they observed significant improvements in exercising cardiac function and cardiac reserve in the exercise group. To this end, they conclude that changes in peak exercise cardiac function, as opposed to resting function, drive changes in VO_2peak_ (Foulkes et al., 2023). Additionally, the lack of change in cardiac function may also be due to the time course of chemotherapy‐induced cardiotoxicity. It is appreciated that drugs such as doxorubicin can have a delayed cardiotoxic response, leading to clear dysfunction and symptomatic disease years after treatment (Zamorano et al., 2016). As such, a longer follow‐up period may yield different results.

### 4.3 Blood pressure

Patients in the MOD group saw significant reductions in central SBP and trended toward a decrease in brachial SBP. This finding was unexpected as HIIT typically leads to greater reductions in blood pressure than MOD in healthy individuals (Angadi et al., 2015; Boutcher & Boutcher, 2017). In this study, patients in both groups saw significant reductions in hemoglobin concentrations. It is known that reduced blood viscosity due to anemia can lead to hypoxic vasodilation and thus decreased systemic vascular resistance (Metivier et al., 2000; Tanimura et al., 2017). Using left ventricular outflow tract velocity time integral and diameter, we estimated cardiac output pre‐ and post‐testing. We observed a significant increase in cardiac output in the HIIT group (4.3 ± 1.1 to 5.3 ± 1.0 L/min; *p* = 0.004) owing to slight increases in both stroke volume and heart rate, and no such increase occurred in the MOD group. Thus, in the face of anemia, those in the HIIT group may have been able to augment cardiac output to maintain blood pressures while those in the MOD group could not.

### 4.4 Biomarkers

Chemotherapy leads to increases in proinflammatory cytokines, which may be related to an increased risk of cardiotoxicity (Ky et al., 2014). In addition, this cardiotoxicity may be reflected by increases in NTproBNP, an important marker for heart failure risk (Vergaro et al., 2021). In the present study, NTproBNP significantly increased within the MOD group, while no changes were observed in the HIIT group. Previous trials have shown similar, albeit mixed, results compared to those found in the present study. Currently, there is limited literature for the ability of NTproBNP/BNP to predict chemotherapy‐related changes in cardiac function (Michel et al., 2020). In a long term follow up of the OptiTrain trial, it was found that those in the exercise groups had lower levels of NTproBNP compared to usual care, and those with higher levels of NTproBNP showed a reduction in VO_2peak_ compared to those with lower levels (Ansund et al., 2021).

### 4.5 Study strengths and limitations

As reported previously (Helms et al., 2026), our study saw a greater than anticipated dropout rate and as a result was statistically underpowered making it hard to appreciate between‐group differences. One limitation to the current study is a lack of true control group which makes it difficult to draw definitive conclusions on the ability of exercise to preserve measures of CVD risk in this population. In addition, due to the use of different exercise modalities for our exercise groups (walking vs. cycling), and use of cycle ergometry for testing, it could be argued that any differences between groups may not be due solely to differences in training intensities, but rather specificity of training. However, the maintenance of heart rate and stroke volume in the face of profound anemia as well as maintenance of NTproBNP in the HIIT group suggest that the findings are unlikely to be due to the modality of testing but due to differences in training interventions.

In addition, while our study was randomized, due to chance all African American participants were in the HIIT group. It has been previously reported that African Americans may have different blood pressure responses following exercise compared to European Americans (Pescatello et al., 2003). While slight differences were noted between ethnicities for blood pressure outcomes in the HIIT group, due to the small sample included it is hard to draw definitive conclusions. Future studies should ensure even distribution of race and ethnicities between groups.

While echocardiography is the standard measure of cardiac function within clinical practice, it has poor test–retest reliability and has limitations in chamber volume estimation (Houard et al., 2021; Jenkins et al., 2004). Use of a more sensitive measure, such as cardiac magnetic resonance (CMR), may lead to different outcomes. In addition, resting measures of cardiac function may not adequately demonstrate changes in systolic function seen during exercise (Foulkes et al., 2023) and, as such, exercise measures of cardiac function should also be obtained.

Further, chemotherapy regimens not traditionally considered to be cardiotoxic were included in the present trial. Previous trials looking at the role of exercise to prevent worsening VO_2peak_ and cardiovascular function during chemotherapy have focused largely on anthracycline therapies (Foulkes et al., 2023; Hornsby et al., 2014; Jones et al., 2013; Kirkham et al., 2017, 2018; Lee et al., 2019; Mijwel et al., 2018). Our trial included both those on immunotherapies (i.e., immune checkpoint inhibitors), as well as other non‐cardiotoxic therapies (i.e., platinum‐based drugs and microtubule inhibitors). The inclusion of broader chemotherapeutic agents allows our results to be more generalizable to breast cancer populations as opposed to just those being treated with anthracyclines. However, this inclusion of less cardiotoxic chemotherapies may have muted our findings.

### 4.6 Future directions

Future research should examine acute and long‐term changes in NTproBNP and other biomarkers and the relationship of these changes to potential declines in VO_2peak_ and cardiac function, following breast cancer treatment. In addition, future studies should compare the efficacy of remotely monitored exercise to supervised exercise training in this population. Given the low usage of supervised rehabilitation in cancer survivors (Hardcastle et al., 2018), it is imperative that effective alternate options are available. Studies should also examine the influence of exercise adherence on the efficacy of outcomes. Additionally, there remains a question about whether there is a minimum amount of exercise required to preserve function, above which there are no additional benefits seen during chemotherapy treatment. Interventions could be designed to examine different exercise amounts/intensities to determine if there is a minimum threshold required for cardio‐protection.

## 5 CONCLUSIONS

In conclusion, this trial found that there were no significant between‐group differences in VO_2peak_ or cardiac function for patients with breast cancer in remotely delivered HIIT and MOD interventions throughout chemotherapy. Adherence to the remote exercise intervention was lower than anticipated (~63% for each group) and may be a limitation to remote interventions. There were no exercise‐related serious adverse events reported in either group, indicating that remotely administered interventions are safe in this population after adequate screening by their physicians. Despite being underpowered, this study is the first to directly compare the effects of different exercise intensities on mitigating cardiotoxicity. Our trial builds on the existing literature indicating that exercise should be prescribed throughout chemotherapy to help offset some of the negative off‐target effects of chemotherapy.

## AUTHOR CONTRIBUTIONS

R.A.K., J.M.Z, and S.S.A. conceived and designed the study. R.A.K., J.M.Z, S.S.A., and A.A. were awarded funding for the study. N.R.W., C.C.E., M.B.M., C.E.H., and Z.S.L. participated in data collection. N.R.W., C.C.E., and M.B.M. analyzed and interpreted the results of the data. N.R.W. drafted the manuscript. Z.B.N., A.A., C.M.B., P.M.D., and T.M. screened and identified patients. All authors edited, revised manuscript, and approved the final version of the manuscript.

## FUNDING INFORMATION

This research was supported by funding within the UVA Comprehensive Cancer Center (P30CA044579) and seed funding from the UVA Imaging and Informatics in Precision Immunomedicine (iPRIME) initiative.

## CONFLICT OF INTEREST STATEMENT

The authors declare no potential conflicts of interest.

## ETHICS STATEMENT

This study was approved by the institutional review board at the University of Virginia and conducted in accordance with the Declaration of Helsinki. All participants provided informed consent prior to any study procedures.

## Supporting information


Table S1.

## Data Availability

Data for this study will be made available upon reasonable request.

## References

[phy270787-bib-0001] American College of Sports Medicine , Liguori, G. , Feito, Y. , Fountaine, C. , & Roy, B. (2021). ACSM's Guidelines for Exercise Testing and Prescription (Eleventh ed.). Wolters Kluwer.

[phy270787-bib-0002] Angadi, S. S. , Bhammar, D. M. , & Gaesser, G. A. (2015). Postexercise hypotension after continuous, aerobic interval, and Sprint interval exercise. Journal of Strength and Conditioning Research, 29(10), 2888–2893. 10.1519/JSC.0000000000000939 25785706

[phy270787-bib-0003] Ansund, J. , Mijwel, S. , Bolam, K. A. , Altena, R. , Wengström, Y. , Rullman, E. , & Rundqvist, H. (2021). High intensity exercise during breast cancer chemotherapy—Effects on long‐term myocardial damage and physical capacity—Data from the OptiTrain RCT. Cardiooncology, 7(1), 7. 10.1186/s40959-021-00091-1 33588948 PMC7883413

[phy270787-bib-0004] Assi, M. , Dufresne, S. , & Rebillard, A. (2020). Exercise shapes redox signaling in cancer. Redox Biology, 35, 101439. 10.1016/j.redox.2020.101439 31974046 PMC7284915

[phy270787-bib-0005] Baba, R. , Nagashima, M. , Goto, M. , Nagano, Y. , Yokota, M. , Tauchi, N. , & Nishibata, K. (1996). Oxygen uptake efficiency slope: A new index of cardiorespiratory functional reserve derived from the relation between oxygen uptake and minute ventilation during incremental exercise. Journal of the American College of Cardiology, 28(6), 1567–1572. 10.1016/s0735-1097(96)00412-3 8917273

[phy270787-bib-0006] Boutcher, Y. N. , & Boutcher, S. H. (2017). Exercise intensity and hypertension: what's new? Journal of Human Hypertension, 31(3), 157–164. 10.1038/jhh.2016.62 27604656

[phy270787-bib-0007] Courneya, K. S. , Segal, R. J. , Mackey, J. R. , Gelmon, K. , Reid, R. D. , Friedenreich, C. M. , Ladha, A. B. , Proulx, C. , Vallance, J. K. , Lane, K. , Yasui, Y. , & McKenzie, D. (2007). Effects of aerobic and resistance exercise in breast cancer patients receiving adjuvant chemotherapy: A multicenter randomized controlled trial. Journal of Clinical Oncology, 25(28), 4396–4404. 10.1200/JCO.2006.08.2024 17785708

[phy270787-bib-0008] Dickinson, J. M. , D'Lugos, A. C. , Mahmood, T. N. , Ormsby, J. C. , Salvo, L. , Dedmon, W. L. , Patel, S. H. , Katsma, M. S. , Mookadam, F. , Gonzales, R. J. , Hale, T. M. , Carroll, C. C. , & Angadi, S. S. (2017). Exercise protects skeletal muscle during chronic doxorubicin administration. Medicine and Science in Sports and Exercise, 49(12), 2394–2403. 10.1249/MSS.0000000000001395 28767526

[phy270787-bib-0009] Foulkes, S. J. , Howden, E. J. , Haykowsky, M. J. , Antill, Y. , Salim, A. , Nightingale, S. S. , Loi, S. , Claus, P. , Janssens, K. , Mitchell, A. M. , Wright, L. , Costello, B. T. , Lindqvist, A. , Burnham, L. , Wallace, I. , Daly, R. M. , Fraser, S. F. , & La Gerche, A. (2023). Exercise for the prevention of anthracycline‐induced functional disability and cardiac dysfunction: The BREXIT study. Circulation, 147(7), 532–545. 10.1161/CIRCULATIONAHA.122.062814 36342348

[phy270787-bib-0010] Given, B. , Given, C. , Azzouz, F. , & Stommel, M. (2001). Physical functioning of elderly cancer patients prior to diagnosis and following initial treatment. Nursing Research, 50(4), 222–232. 10.1097/00006199-200107000-00006 11480531

[phy270787-bib-0011] Hardcastle, S. J. , Maxwell‐Smith, C. , Kamarova, S. , Lamb, S. , Millar, L. , & Cohen, P. A. (2018). Factors influencing non‐participation in an exercise program and attitudes towards physical activity amongst cancer survivors. Supportive Care in Cancer, 26(4), 1289–1295. 10.1007/s00520-017-3952-9 29090387

[phy270787-bib-0012] Helms, C. E. , Krukowski, R. A. , Weeldreyer, N. R. , Glick, J. , Ellison, C. C. , Mabalot, M. B. , Day, K. R. , Millard, T. , Dillon, P. M. , Brenin, C. M. , Angadi, S. S. , & Zoellner, J. M. (2026). A pilot RCT of two remotely monitored exercise interventions in breast cancer patients receiving cytotoxic chemotherapy. Translational Journal of the American College of Sports Medicine, 11(1). https://journals.lww.com/acsm‐tj/fulltext/2026/01160/a_pilot_rct_of_two_remotely_monitored_exercise.9.aspx

[phy270787-bib-0014] Hornsby, W. E. , Douglas, P. S. , West, M. J. , Kenjale, A. A. , Lane, A. R. , Schwitzer, E. R. , Ray, K. A. , Herndon, J. E., 2nd , Coan, A. , Gutierrez, A. , Hornsby, K. P. , Hamilton, E. , Wilke, L. G. , Kimmick, G. G. , Peppercorn, J. M. , & Jones, L. W. (2014). Safety and efficacy of aerobic training in operable breast cancer patients receiving neoadjuvant chemotherapy: A phase II randomized trial. Acta Oncologica, 53(1), 65–74. 10.3109/0284186X.2013.781673 23957716

[phy270787-bib-0015] Houard, L. , Militaru, S. , Tanaka, K. , Pasquet, A. , Vancraeynest, D. , Vanoverschelde, J. L. , Pouleur, A. C. , & Gerber, B. L. (2021). Test‐retest reliability of left and right ventricular systolic function by new and conventional echocardiographic and cardiac magnetic resonance parameters. European Heart Journal Cardiovascular Imaging, 22(10), 1157–1167. 10.1093/ehjci/jeaa206 32793957

[phy270787-bib-0016] Jacquinot, Q. , Meneveau, N. , Falcoz, A. , Bouhaddi, M. , Roux, P. , Degano, B. , Chatot, M. , Curtit, E. , Mansi, L. , Paillard, M. J. , Bazan, F. , Chaigneau, L. , Dobi, E. , Meynard, G. , Vernerey, D. , Pivot, X. , & Mougin, F. (2022). Cardiotoxicity is mitigated after a supervised exercise program in HER2‐positive breast cancer undergoing adjuvant trastuzumab. Frontiers in Cardiovascular Medicine, 9, 1000846. 10.3389/fcvm.2022.1000846 36211552 PMC9537598

[phy270787-bib-0017] Jenkins, C. , Bricknell, K. , Hanekom, L. , & Marwick, T. H. (2004). Reproducibility and accuracy of echocardiographic measurements of left ventricular parameters using real‐time three‐dimensional echocardiography. Journal of the American College of Cardiology, 44(4), 878–886. 10.1016/j.jacc.2004.05.050 15312875

[phy270787-bib-0018] Jones, L. W. , Fels, D. R. , West, M. , Allen, J. D. , Broadwater, G. , Barry, W. T. , Wilke, L. G. , Masko, E. , Douglas, P. S. , Dash, R. C. , Povsic, T. J. , Peppercorn, J. , Marcom, P. K. , Blackwell, K. L. , Kimmick, G. , Turkington, T. G. , & Dewhirst, M. W. (2013). Modulation of circulating angiogenic factors and tumor biology by aerobic training in breast cancer patients receiving neoadjuvant chemotherapy. Cancer Prevention Research (Philadelphia, Pa.), 6(9), 925–937. 10.1158/1940-6207.CAPR-12-0416 23842792 PMC3800005

[phy270787-bib-0019] Kavazis, A. N. , Smuder, A. J. , Min, K. , Tumer, N. , & Powers, S. K. (2010). Short‐term exercise training protects against doxorubicin‐induced cardiac mitochondrial damage independent of HSP72. American Journal of Physiology. Heart and Circulatory Physiology, 299(5), H1515–H1524. 10.1152/ajpheart.00585.2010 20833957 PMC2993189

[phy270787-bib-0020] Kirkham, A. A. , Beaudry, R. I. , Paterson, D. I. , Mackey, J. R. , & Haykowsky, M. J. (2019). Curing breast cancer and killing the heart: A novel model to explain elevated cardiovascular disease and mortality risk among women with early stage breast cancer. Progress in Cardiovascular Diseases, 62(2), 116–126. 10.1016/j.pcad.2019.02.002 30797800

[phy270787-bib-0021] Kirkham, A. A. , Eves, N. D. , Shave, R. E. , Bland, K. A. , Bovard, J. , Gelmon, K. A. , Virani, S. A. , McKenzie, D. , Stöhr, E. J. , Waburton, D. E. R. , & Campbell, K. L. (2018). The effect of an aerobic exercise bout 24 h prior to each doxorubicin treatment for breast cancer on markers of cardiotoxicity and treatment symptoms: A RCT. Breast Cancer Research and Treatment, 167(3), 719–729. 10.1007/s10549-017-4554-4 29110150

[phy270787-bib-0022] Kirkham, A. A. , Shave, R. E. , Bland, K. A. , Bovard, J. M. , Eves, N. D. , Gelmon, K. A. , McKenzie, D. , Virani, S. A. , Stöhr, E. J. , Warburton, D. E. R. , & Campbell, K. L. (2017). Protective effects of acute exercise prior to doxorubicin on cardiac function of breast cancer patients: A proof‐of‐concept RCT. International Journal of Cardiology, 245, 263–270. 10.1016/j.ijcard.2017.07.037 28735755

[phy270787-bib-0023] Kwakman, J. J. M. , Elshot, Y. S. , Punt, C. J. A. , & Koopman, M. (2020). Management of cytotoxic chemotherapy‐induced hand‐foot syndrome. Oncology Reviews, 14(1), 442. 10.4081/oncol.2020.442 32431787 PMC7232019

[phy270787-bib-0024] Ky, B. , Putt, M. , Sawaya, H. , French, B. , Januzzi, J. L., Jr. , Sebag, I. A. , Plana, J. C. , Cohen, V. , Banchs, J. , Carver, J. R. , Wiegers, S. E. , Martin, R. P. , Picard, M. H. , Gerszten, R. E. , Halpern, E. F. , Passeri, J. , Kuter, I. , & Scherrer‐Crosbie, M. (2014). Early increases in multiple biomarkers predict subsequent cardiotoxicity in patients with breast cancer treated with doxorubicin, taxanes, and trastuzumab. Journal of the American College of Cardiology, 63(8), 809–816. 10.1016/j.jacc.2013.10.061 24291281 PMC4286181

[phy270787-bib-0025] Lee, K. , Kang, I. , Mack, W. J. , Mortimer, J. , Sattler, F. , Salem, G. , Lu, J. , & Dieli‐Conwright, C. M. (2019). Effects of high‐intensity interval training on vascular endothelial function and vascular wall thickness in breast cancer patients receiving anthracycline‐based chemotherapy: A randomized pilot study. Breast Cancer Research and Treatment, 177(2), 477–485. 10.1007/s10549-019-05332-7 31236810 PMC6661195

[phy270787-bib-0026] Lenneman, C. G. , & Sawyer, D. B. (2016). Cardio‐oncology: An update on cardiotoxicity of cancer‐related treatment. Circulation Research, 118(6), 1008–1020. 10.1161/CIRCRESAHA.115.303633 26987914

[phy270787-bib-0027] MacInnis, M. J. , & Gibala, M. J. (2017). Physiological adaptations to interval training and the role of exercise intensity. The Journal of Physiology, 595(9), 2915–2930. 10.1113/JP273196 27748956 PMC5407969

[phy270787-bib-0028] Mclaughlin, M. , Florida‐James, G. , & Ross, M. (2021). Breast cancer chemotherapy vascular toxicity: A review of mediating mechanisms and exercise as a potential therapeutic. Vascular Biology, 2021(1), R106–R120. 10.1530/vb-21-0013 PMC8630759 34870095

[phy270787-bib-0029] Metivier, F. , Marchais, S. J. , Guerin, A. P. , Pannier, B. , & London, G. M. (2000). Pathophysiology of anaemia: Focus on the heart and blood vessels. Nephrology, Dialysis, Transplantation, 15(3), 14–18. 10.1093/oxfordjournals.ndt.a027970 11032352

[phy270787-bib-0030] Michel, L. , Mincu, R. I. , Mahabadi, A. A. , Settelmeier, S. , al‐Rashid, F. , Rassaf, T. , & Totzeck, M. (2020). Troponins and brain natriuretic peptides for the prediction of cardiotoxicity in cancer patients: A meta‐analysis. European Journal of Heart Failure, 22(2), 350–361. 10.1002/ejhf.1631 31721381

[phy270787-bib-0031] Mijwel, S. , Backman, M. , Bolam, K. A. , Olofsson, E. , Norrbom, J. , Bergh, J. , Sundberg, C. J. , Wengström, Y. , & Rundqvist, H. (2018). Highly favorable physiological responses to concurrent resistance and high‐intensity interval training during chemotherapy: The OptiTrain breast cancer trial. Breast Cancer Research and Treatment, 169(1), 93–103. 10.1007/s10549-018-4663-8 29349712 PMC5882634

[phy270787-bib-0032] Mitchell, C. , Rahko, P. S. , Blauwet, L. A. , Canaday, B. , Finstuen, J. A. , Foster, M. C. , Horton, K. , Ogunyankin, K. O. , Palma, R. A. , & Velazquez, E. J. (2019). Guidelines for performing a comprehensive transthoracic echocardiographic examination in adults: Recommendations from the American Society of Echocardiography. Journal of the American Society of Echocardiography, 32(1), 1–64. 10.1016/j.echo.2018.06.004 30282592

[phy270787-bib-0033] Myers, J. , Arena, R. , Dewey, F. , Bensimhon, D. , Abella, J. , Hsu, L. , Chase, P. , Guazzi, M. , & Peberdy, M. A. (2008). A cardiopulmonary exercise testing score for predicting outcomes in patients with heart failure. American Heart Journal, 156(6), 1177–1183. 10.1016/j.ahj.2008.07.010 19033016

[phy270787-bib-0034] Nagueh, S. F. , Smiseth, O. A. , Appleton, C. P. , Byrd, B. F., 3rd , Dokainish, H. , Edvardsen, T. , Flachskampf, F. A. , Gillebert, T. C. , Klein, A. L. , Lancellotti, P. , Marino, P. , Oh, J. K. , Popescu, B. A. , & Waggoner, A. D. (2016). Recommendations for the evaluation of left ventricular diastolic function by echocardiography: An update from the American Society of Echocardiography and the European Association of Cardiovascular Imaging. Journal of the American Society of Echocardiography, 29(4), 277–314. 10.1016/j.echo.2016.01.011 27037982

[phy270787-bib-0035] National Cancer Institute . (2025). SEER. Cancer stat facts: Female breast cancer. https://seer.cancer.gov/statfacts/html/breast.html

[phy270787-bib-0036] Paramita, N. , Puspasari, B. C. , Arrody, R. , Kartinah, N. T. , Andraini, T. , Mardatillah, J. , Rusli, H. , & Santoso, D. I. S. (2022). Protective effect of high‐intensity interval training (HIIT) and moderate‐intensity continuous training (MICT) against vascular dysfunction in hyperglycemic rats. Journal of Nutrition and Metabolism, 2022, 5631488. 10.1155/2022/5631488 36510592 PMC9741543

[phy270787-bib-0037] Patnaik, J. L. , Byers, T. , DiGuiseppi, C. , Dabelea, D. , & Denberg, T. D. (2011). Cardiovascular disease competes with breast cancer as the leading cause of death for older females diagnosed with breast cancer: A retrospective cohort study. Breast Cancer Research, 13(3), R64. 10.1186/bcr2901 21689398 PMC3218953

[phy270787-bib-0038] Pescatello, L. S. , Bairos, L. , Vanheest, J. L. , Maresh, C. M. , Rodriguez, N. R. , Moyna, N. M. , DiPasquale, C. , Collins, V. , Meckes, C. L. , Krueger, L. , & Thompson, P. D. (2003). Postexercise hypotension differs between white and black women. American Heart Journal, 145(2), 364–370. 10.1067/mhj.2003.107 12595857

[phy270787-bib-0039] Schmid, P. , Cortes, J. , Pusztai, L. , McArthur, H. , Kümmel, S. , Bergh, J. , Denkert, C. , Park, Y. H. , Hui, R. , Harbeck, N. , Takahashi, M. , Foukakis, T. , Fasching, P. A. , Cardoso, F. , Untch, M. , Jia, L. , Karantza, V. , Zhao, J. , Aktan, G. , … O'Shaughnessy, J. (2020). Pembrolizumab for early triple‐negative breast cancer. The New England Journal of Medicine, 382(9), 810–821. 10.1056/NEJMoa1910549 32101663

[phy270787-bib-0040] Society AC . (2025). Cancer facts & figures 2025. https://www.cancer.org/research/cancer‐facts‐statistics/all‐cancer‐facts‐figures/2025‐cancer‐facts‐figures.html

[phy270787-bib-0041] Speck, R. M. , Courneya, K. S. , Masse, L. C. , Duval, S. , & Schmitz, K. H. (2010). An update of controlled physical activity trials in cancer survivors: A systematic review and meta‐analysis. Journal of Cancer Survivorship, 4(2), 87–100. 10.1007/s11764-009-0110-5 20052559

[phy270787-bib-0042] Sweeney, C. , Schmitz, K. H. , Lazovich, D. , Virnig, B. A. , Wallace, R. B. , & Folsom, A. R. (2006). Functional limitations in elderly female cancer survivors. Journal of the National Cancer Institute, 98(8), 521–529. 10.1093/jnci/djj130 16622121

[phy270787-bib-0043] Tanimura, M. , Dohi, K. , Fujimoto, N. , Moriwaki, K. , Omori, T. , Sato, Y. , Sugiura, E. , Kumagai, N. , Nakamori, S. , Kurita, T. , Fujii, E. , Yamada, N. , & Ito, M. (2017). Effect of anemia on cardiovascular hemodynamics, therapeutic strategy and clinical outcomes in patients with heart failure and hemodynamic congestion. Circulation Journal, 81(11), 1670–1677. 10.1253/circj.CJ-17-0171 28626160

[phy270787-bib-0044] Thijssen, D. H. J. , Bruno, R. M. , van Mil, A. , Holder, S. M. , Faita, F. , Greyling, A. , Zock, P. L. , Taddei, S. , Deanfield, J. E. , Luscher, T. , Green, D. J. , & Ghiadoni, L. (2019). Expert consensus and evidence‐based recommendations for the assessment of flow‐mediated dilation in humans. European Heart Journal, 40(30), 2534–2547. 10.1093/eurheartj/ehz350 31211361

[phy270787-bib-0045] Tucker, W. J. , Jarrett, C. L. , D'Lugos, A. C. , Angadi, S. S. , & Gaesser, G. A. (2021). Effects of indulgent food snacking, with and without exercise training, on body weight, fat mass, and cardiometabolic risk markers in overweight and obese men. Physiological Reports, 9(22), e15118. 10.14814/phy2.15118 34816612 PMC8611507

[phy270787-bib-0046] Vergaro, G. , Gentile, F. , Meems, L. M. G. , Aimo, A. , Januzzi, J. L., Jr. , Richards, A. M. , Lam, C. S. P. , Latini, R. , Staszewsky, L. , Anand, I. S. , Cohn, J. N. , Ueland, T. , Gullestad, L. , Aukrust, P. , Brunner‐la Rocca, H. P. , Bayes‐Genis, A. , Lupón, J. , Yoshihisa, A. , Takeishi, Y. , … Emdin, M. (2021). NT‐proBNP for risk prediction in heart failure: Identification of optimal cutoffs across body mass index categories. JACC Heart Failure, 9(9), 653–663. 10.1016/j.jchf.2021.05.014 34246607

[phy270787-bib-0047] Wisloff, U. , Ellingsen, O. , & Kemi, O. J. (2009). High‐intensity interval training to maximize cardiac benefits of exercise training? Exercise and Sport Sciences Reviews, 37(3), 139–146. 10.1097/JES.0b013e3181aa65fc 19550205

[phy270787-bib-0048] Yang, H. , Villani, R. M. , Wang, H. , Simpson, M. J. , Roberts, M. S. , Tang, M. , & Liang, X. (2018). The role of cellular reactive oxygen species in cancer chemotherapy. Journal of Experimental & Clinical Cancer Research, 37(1), 266. 10.1186/s13046-018-0909-x 30382874 PMC6211502

[phy270787-bib-0049] Zamorano, J. L. , Lancellotti, P. , Rodriguez Munoz, D. , Aboyans, V. , Asteggiano, R. , Galderisi, M. , Habib, G. , Lenihan, D. J. , Lip, G. Y. H. , Lyon, A. R. , Lopez Fernandez, T. , Mohty, D. , Piepoli, M. F. , Tamargo, J. , Torbicki, A. , & Suter, T. M. (2016). 2016 ESC position paper on cancer treatments and cardiovascular toxicity developed under the auspices of the ESC Committee for practice guidelines: The task force for cancer treatments and cardiovascular toxicity of the European Society of Cardiology (ESC). European Heart Journal, 37(36), 2768–2801. 10.1093/eurheartj/ehw211 27567406

[phy270787-bib-0050] Zhang, Y. , Wang, Y. , Shi, J. , Hua, Z. , & Xu, J. (2019). Cardiac output measurements via echocardiography versus thermodilution: A systematic review and meta‐analysis. PLoS One, 14(10), e0222105. 10.1371/journal.pone.0222105 31581196 PMC6776392

