# Supplementary material for: The effect of two remote exercise programs on cardiorespiratory fitness, cardiac function, and vascular health in patients with breast cancer
Source: Physiol Rep. 2026 Feb 17;14(4):e70787. doi: 10.14814/phy2.70787 (PMC12914078; doi:10.14814/phy2.70787)
Supplement: Supplementary file 1 — Table S1. [file PHY2-14-e70787-s001.pdf]

Supplementary Table 1. Blood markers and measures of inflammation and cardiac damage.

	MOD			HIIT			Between group
	Pre	Post	*P*	Pre	Post	*p*	*p*
IL-6 (pg/mL)[a]	11.0 ± 11.0	28.9 ± 62.4	0.917	6.5 ± 7.4	4.0 ± 3.5	0.500	0.855
IL-1β (pg/mL)	1.2 ± 1.5	1.6 ± 1.9	0.716	1.1 ± 1.4	0.40 ± 0.14	0.397	0.428
CRP (mg/L)[a]	10.2 ± 8.7	84.2 ± 156.4	0.128	27.4 ± 19.8	138.2 ± 204.3	0.116	0.935
TNF-α (pg/mL)	3.6 ± 1.6	4.5 ± 2.1	0.220	5.2 ± 3.5	4.3 ± 1.0	0.588	0.268
NTproBNP (pg/mL)	104.3 ± 35.1	158.1 ± 56.0	0.018*	178.6 ± 49.9	171.1 ± 49.5	0.793	0.075

* Indicates $p < 0.05$. [a] Indicates nonparametric test used. IL, interleukin; CRP, C-Reactive protein; TNF-α, Tumor necrosis factor alpha; TnI, Troponin I; NTproBNP, N-terminal pro-brain natriuretic peptide.
